# Determinants of the plasma metabolome: cross-sectional and longitudinal associations over six years in the NESDA cohort

**DOI:** 10.1016/j.ebiom.2026.106384

**Published:** 2026-07-23

**Authors:** Dennis Klose, Yuri Milaneschi, Laura K.M. Han, Nic J.A. van der Wee, Matthias Arnold, Christopher R. Brydges, Siamak Mahmoudian Dehkordi, Gabi Kastenmüller, Rima Kaddurah Daouk, Brenda W.J.H. Penninx

**Affiliations:** a Department of Psychiatry, Amsterdam University Medical Center, Vrije Universiteit Amsterdam, Amsterdam, the Netherlands; b Personalized Medicine and Mental Health Programs, Amsterdam University Medical Center, Amsterdam Public Health Research Institute, Amsterdam, the Netherlands; c Mood, Anxiety, Psychosis, Sleep & Stress Program and Complex Trait Genetics Program, Amsterdam University Medical Center, Amsterdam Neuroscience Research Institute, Amsterdam, the Netherlands; d Department of Psychiatry and Psychology, Leiden University Medical Center, Universiteit Leiden, Leiden, the Netherlands; e Institute of Computational Biology, Helmholtz Zentrum München - German Research Center for Environmental Health, Neuherberg, Germany; f Department of Psychiatry and Behavioral Sciences, Duke University, Durham, NC, USA; g Duke Institute of Brain Sciences, Duke University, Durham, NC, USA; h Department of Medicine, Duke University, Durham, NC, USA

**Keywords:** Plasma, Metabolomics, Lifestyle, Stress, Longitudinal study, Epidemiology

## Abstract

**Background:**

The plasma metabolome represents a valuable molecular readout of a person’s physiological state, yet its relation to health, stress and lifestyle remains underexplored collectively.

**Methods:**

Here, we conducted an untargeted metabolomics analysis using 3804 paired samples from 1902 participants of the observational Netherlands Study of Depression and Anxiety at baseline and six-year follow-up, quantifying 680 plasma metabolites. We characterised five metabolome principal components, three with distinct biochemical enrichments related to transmembrane transport, sphingolipid, and amino acid metabolism.

**Findings:**

Metabolite levels showed moderate intrapersonal correlation between baseline and six-year follow-up (ICC_median_ = 0.482), and 22% of metabolites showed standardised mean differences >0.2. Multivariate linear modelling on 18 baseline determinants across demographics, psychosocial environment, lifestyle, somatic and mental health explained a maximum of 35% of baseline metabolome PC variance and 12% of six-year change ΔPC variance. Demographic (e.g., sex, age), somatic health (e.g., BMI, medication) and lifestyle factors (e.g., smoking, alcohol intake) demonstrated strong associations both cross-sectionally and longitudinally, while psychosocial factors and mental health contributed minor explained variance in comparison.

**Interpretation:**

Altogether, our study provides hierarchical insights into the cross-sectional and longitudinal implications of health, stress, and lifestyle exposures for the plasma metabolome.

**Funding:**

Geestkracht program of the Netherlands Organisation for Health Research and Development, the Dutch Research Council and the Dutch Ministry of Education, Culture and Science, Stress in Action, Amsterdam Neuroscience, ImmunoMIND, the National Institute of Mental Health, National Institute on Ageing and the Foundation for the National Institutes of Health.

**Research in context**

**Evidence before this study**

Longitudinal metabolomic studies with detailed phenotyping of study participants are rare and have mostly focused on the cross-sectional relation between metabolite levels and genetics, diet, the microbiome, and clinical factors. Psychosocial determinants, on the other hand, are understudied. Although previous research has demonstrated correlations between psychosocial indicators such as stress or depression with metabolic alterations in humans, most studies were limited by small sample sizes, narrow metabolite coverage, or loosely defined stress exposures. Further, it remains unclear how their magnitude compares to other exposures such as demographics or somatic and lifestyle indicators. In addition, longitudinal studies examining metabolic change trajectories are limited and use varying matrices (serum/plasma) or analysis techniques (NMR/LC-MS). Therefore, we do not have a detailed understanding of the stability of metabolites over multiple years, and to what extent demographic, somatic health, lifestyle, mental health, and psychosocial determinants drive metabolomic change.

**Added value of this study**

We collectively examined 18 determinants spanning five major domains–demographics, psychosocial environment, lifestyle, somatic health, and mental health–and their associations with 680 plasma metabolites expressed as five principal components in 1902 participants from the NESDA cohort at two timepoints (baseline and six-year follow-up). Using the same high-throughput LC-MS technology, we assessed both cross-sectional and longitudinal metabolomic variance associated with each domain. This comprehensive approach enabled direct comparison of how strongly different life exposures–from psychosocial stressors (work stress, daily stress, childhood trauma) to lifestyle factors (smoking, alcohol, physical activity) and health indicators (BMI, medication, chronic conditions)–are associated with changes to the plasma metabolome both at a single timepoint and over six years.

**Implications of all the available evidence**

We identified several biochemical pathways linked to distinct determinants across all five exposure domains, suggesting a hierarchy of influences on the metabolome: Demographic factors (sex, age), somatic health indicators (BMI, medication use, chronic conditions), and lifestyle behaviours (smoking, alcohol intake) demonstrated the strongest associations both cross-sectionally and longitudinally. Psychosocial factors and mental health, while showing some statistically significant metabolomic associations, contributed comparatively modest explained variance. While we have shown that the general plasma metabolome composition stays mostly stable over six years in our sample (confirming previous reports), several influences like education level, medication use, work stress, daily stress, and smoking are associated with changes to metabolite stability over time. These findings provide a framework for understanding how different exposure domains collectively shape the plasma metabolome, and may help prioritise future mechanistic and intervention studies.

## Introduction

The plasma metabolome represents the collection of exogenous chemicals and endogenous, small molecules originating from active metabolic processes, consisting of diverse compounds which facilitate energy uptake and storage, support cellular communication and maintain structural integrity.^1^ While several key influences on the blood metabolome composition have been identified in previous analyses using serum or plasma–including sex, age, genetics, diet, the gut microbiome, anthropometrics, circadian rhythm, diseases and persistent organic pollutants^2^, ^3^, ^4^, ^5^, ^6^, ^7^–many sources of variation remain unexplored. Importantly, existing research has examined these influences in isolation, leaving a significant gap in our understanding of how multiple diverse factors contribute collectively. Metabolic alterations may play a fundamental role in the development of somatic and mental illnesses and can often be associated with environmental exposures such as medication, pollutants, lifestyle choices or psychological stressors.^8^, ^9^, ^10^, ^11^, ^12^, ^13^, ^14^, ^15^, ^16^, ^17^, ^18^ While the metabolome-wide influence of psychological stressors and mental health has been investigated in previous studies,^3^^,^^13^ the role of more varied and detailed psychosocial factors in the context of other exposures has not been thoroughly investigated so far.

Here, we present a large-scale LC-MS-based study (n = 1902) examining the associations of 18 determinants with 680 identified human plasma metabolites leveraging both cross-sectional and longitudinal data from six-year follow-up assessments (FU6), overcoming limitations of previous studies pertaining to sample size, assessed determinants and timepoints. Our primary objective is to estimate the percentage of variance explained by determinants spanning five conceptual domains (demographics, psychosocial environment, lifestyle, somatic, i.e., physical, health and mental health) in metabolite levels measured cross-sectionally and longitudinally focusing on change over six years. For this, we derive five distinct metabolome principal components and characterise them based on their potential enrichment in biochemical processes and subsequently use these to establish metabolome baseline scores and metabolome change scores for linear modelling. Metabolomic change, also referred to as metabotype instability in previous literature, has been identified as a promising marker of various disease onsets, cardiovascular conditions and increased mortality.^6^^,^^19^^,^^20^

Our study builds on and extends two previous key studies. Bar et al. (2020) assessed determinants of the human serum metabolome in a healthy cohort with limited drug intake, precluding a comparison with somatic or mental illness influences.^3^ Despite measuring more metabolites (n = 1251), their sample was smaller (n = 491) and cross-sectional. Chen et al. (2022) similarly estimated explained variance of key demographic and lifestyle factors on the plasma metabolome (n = 1183), additionally incorporating genetics, diet, and microbiome composition, and examined metabolite-specific stability over 4 years.^4^ However, their longitudinal sample was limited to 311 individuals. The present study complements both by combining a large sample with extensive phenotyping across two timepoints, including clinical populations, and by introducing a metabotype-level approach to characterise temporal metabolome stability, offering a more holistic perspective than single-metabolite analyses.

Altogether, this study gives an overarching and hierarchical overview into which baseline characteristics and life experiences are embedded into the plasma metabolome on two timescales.

## Methods

### Study design and participants

Data are from the Netherlands Study of Depression and Anxiety (NESDA), an ongoing multi-centre, observational, naturalistic, and longitudinal cohort study examining the course and consequences of depressive and anxiety disorders. A detailed description of the study rationale, design and methods is given elsewhere.^21^ Briefly, 2981 adults (18–65 years old) with or without a diagnosis of depression and/or anxiety disorder were recruited from community samples, primary care practices, and mental health organisations between 2004 and 2007. Detailed assessments were repeated after one, two, four and six years of follow-up. Exclusion criteria were being diagnosed with a current clinically overt psychiatric disorder other than depression and anxiety as well as lacking proficiency in Dutch.

### Ethics

Ethical approval was obtained centrally from the Ethical Review Board of the Vrije Universiteit Medical Centre (reference number 2003/183) in Amsterdam, the Netherlands, as well as from the Ethics Review Boards of the other participating research centres. All participants provided written informed consent.

### Metabolomic data generation and preprocessing

Plasma metabolite measurement and subsequent data pre-processing have been described elsewhere extensively^9^ (for further details see Supplementary Information). Data was analysed and pre-processed in line with protocols adopted by established international consortia (https://www.omicscience.org/,^22^, ^23^, ^24^), log_2_-transformed and winsorised. In brief, fasting EDTA plasma samples were collected in the morning at baseline and FU6 and were stored at −80 °C. Samples were sent for analysis where metabolome profiles were assessed using the mass spectrometry-based untargeted HD4 platform from Metabolon Inc. (Durham, NC, USA), with participant-paired samples being processed on the same plate within the same batch. Shipment was performed in two batches. In total, 4827 samples were measured, 2812 from baseline and 2015 from FU6. From that, 1902 study participants had available data across the two timepoints. From 820 measured metabolites in total, 139 unknown metabolites and the anti-clotting agent EDTA were excluded. Thus, 680 metabolites were available for analysis. Mutually exclusive labels provided by Metabolon Inc. were used to categorise metabolite classes: lipids, amino acids, xenobiotics, peptides, cofactors and vitamins, nucleotides, carbohydrates, partially characterised molecules, and energy.

### Clinical assessments and other measures at baseline

In total, 18 exposome factors (determinants) from five conceptual domains were selected based on their known general relevance for human metabolism, physiology and/or their influence on experiencing psychological stress.^25^, ^26^, ^27^, ^28^, ^29^, ^30^, ^31^, ^32^, ^33^, ^34^, ^35^, ^36^ Of note, we did not observe concerning levels of multicollinearity across our chosen determinants (max. Pearson’s *r* = 0.48 for N somatic diseases ∼ N frequent medication, Supplementary Fig. S4). A missing data analysis related to the full model is provided in Supplementary Information.

#### Demographics

*Sex, age*, and *years of education* were assessed via basic intake questionnaires at study start. The *degree of urbanisation* was determined based on current zip code and population density information.

#### Psychosocial environment

*Partner status* was assessed via a basic intake questionnaire at study start. *Work related stress* was assessed using the Karasek questionnaire,^37^ with high strain work being defined as current work with self-reported high job demand scores (≥0.5) and low job control scores (≤0.5). Job demand refers to the expected pace of work, workload, and work intensity whereas job control assesses self-perceived autonomy at the workplace. The *leisure activity score* is a summed score of five items from a questionnaire asking about the current frequency of social free time activities such as going to a bar, museum, restaurant, association, amusement park, cinema, club, or sports activity outside of home. The *daily hassles score* was derived using a questionnaire asking about daily life social stressors such as interpersonal difficulties or conflicts with friends, colleagues or family, high mental load, rejection, disappointment, or financial struggles in the past month. The reported *childhood trauma score* was assessed using the Childhood Trauma Questionnaire from the NEMESIS study^38^ and asked about traumatic experiences such as parental divorce, juvenile prison detention, foster family or child home placement, emotional neglect and psychological, physical or sexual abuse in the first 16 years of life. The *number of negative recent life events* was determined based on an abridged version of the Brugha questionnaire^39^ which asks about the occurrence of adverse events in the past year, such as death or serious illness of loved ones, job loss, terminated relationship or friendship, contact with the police or court and other events.

#### Lifestyle

*Alcohol intake* was calculated based on data originating from the AUDIT questionnaire and is expressed as drinks per week.^40^ *Smoking status* was determined based on data originating from the Fagerström questionnaire and is expressed as never smoker, former smoker and current smoker.^41^ *Physical activity* was assessed using the International physical activity questionnaire and is expressed as MET-minutes per week,^42^ capturing sports activity, movement, and commuting.

#### Somatic health

We deduced the *number of somatic diseases* of participants based on a questionnaire asking about the following current, chronic conditions: asthma, heart disease, diabetes, stroke, arthritis, cancer, hypertension, intestinal disorders, liver disease, epilepsy, chronic fatigue, allergies, thyroid gland disease, injury in past year, head injury (ever), and others. *Number of medications* was defined as drugs taken on ≥50% of the days in a current month for conditions falling under a WHO ATC classification. Dietary and homoeopathic supplements were excluded. The top 10 most frequently used medications in our sample were paracetamol, ibuprofen, paroxetine, oxazepam, omeprazole, metoprolol, citalopram, venlafaxine, amiloride and simvastatin. For the frequency of ATC codes in our sample see Supplementary Table S1. *BMI* was determined as current body weight [kg]/height^2^ [m].

#### Mental health

The *depression diagnosis* variable in this study encompasses CIDI diagnosed major depressive disorder or dysthymia in the past 6 months. The *anxiety diagnosis* encompasses CIDI diagnosed generalised anxiety disorder, agoraphobia, panic disorder, or social phobia in the past 6 months.

### Statistics

If not stated otherwise, all analyses were conducted in R (version 4.3.3) in Microsoft VS Code. See Supplementary Information for further details on packages used for analysis and plotting (excl. dependencies).

#### Intraclass correlation

We assessed intraclass correlations between baseline and FU6 for 680 metabolites to assess within-subject change using the ICC function from the R psych package. We reported ICC1; ICC3 gave similar results (median = 0.496).

#### Principal component analysis

We performed principal component analysis on a data matrix of 3804 samples from baseline and FU6 × 680 metabolites features using the prcomp function from base R. The scale and centre arguments were set to TRUE. We calculated a metabolome change score per PC for each sample as ΔPC = PC_FU6_ − PC_Baseline_. To perform a significance test for principal components themselves and the respective loadings for each principal component, we used the PCAtest function from the PCAtest R package.^43^ All PCs were significant, while only a subset of metabolites represented significant loadings for each PC. Significance was determined based on 100 permutations × 100 bootstraps of the original data matrix. For internal validation of the PCA results (scree plot and loadings) this was repeated for 2812−1902 = 910 additional baseline samples, including NESDA study participants, 351 of which were siblings of main NESDA participants.

#### Metabolite IDs

For each metabolite, Metabolon Inc. provided the chemical name, its PubChem ID and its HMDB ID.^44^^,^^45^ PubChem IDs had lower missingness than HMDB IDs and were thus used for metabolite set enrichment analysis (MSEA). For gene ontology analyses (GO), protein interaction partners of metabolites per PC1-5 were used as input and expressed as gene symbols. The HMDB API was used to derive those protein interaction partners, requiring HMDB IDs as queries. Therefore, different IDs were used compared to MSEA. In cases where multiple IDs were available per metabolite as provided by Metabolon Inc., the first one listed was chosen.

#### Metabolite set enrichment analysis

To identify enrichments of certain functional pathways along a metabolite list (ranked by metabolite loading score per PC from highest to lowest), we used the WebGestaltR function of the WebGestaltR package, setting enrichMethod to “GSEA”, organism to “hsapiens“, enrichDatabase to “pathway_RampDB_Metabolomics “, interestGeneType to “pubchem” and otherwise standard settings.^46^ Background metabolite selection is not needed for GSEA. Higher normalised enrichment scores indicate enrichment of metabolites closer to the top of the list, where the most important metabolites for the respective PC are found. WGCNA/ORA analysis details are provided in Supplementary Information.

#### Gene ontology analysis

To identify significant overlaps between metabolite protein interaction partners per PC and Kyoto Encyclopaedia of Genes and Genomes (KEGG) pathway sets, we used the ShinyGO v0.82 website https://bioinformatics.sdstate.edu/go/^47^ with “Use pathway DB for gene counts” set to TRUE, showing the top 10 enriched pathways with standard settings. KEGG was chosen to investigate general human biochemical pathways.^48^

#### Linear regression

To model PC-baseline and ΔPC scores (outcomes) on baseline determinants (predictors), we used the lm function from base R. Shipment batch was included as covariate for both outcomes, the respective PC-baseline score was included as a covariate for all ΔPC models. Outcomes and all numerical predictors were scaled (“robust scaling” method, using the ratio of median and interquartile range). *Degree of urbanisation* was an ordinal variable. *Smoking status* and *work-related* *stress* were categorical variables. *Sex*, *partner status*, *depression diagnosis*, and *anxiety diagnosis* were dichotomous variables. All other variables were numeric. To compare the models, R^2^ coefficients of determination were adjusted for the number of predictors in the model. Sample sizes per model differed depending on missingness of data for some determinants. Sensitivity analyses restricted to participants with complete data (n = 1648) yielded comparable results (correlations with original adj. R^2^: Pearson’s *r* = 0.999 for PC-baseline models, *r* = 0.995 for ΔPC models), confirming our findings’ robustness. Nominal *P*-values were corrected using the BH method per linear model.^49^ As part of the longitudinal analysis (which estimates the associations between baseline exposures and subsequent change in metabolome PCs), we made use of a base model in addition to the five domain models, which included only the respective PC-baseline score and the shipment covariate. This ensures that we quantify the variance of metabolome change scores solely explained by baseline metabolome PCs and technical confounding to understand the additional explained variance coming from the determinants of interest in the other models. Therefore, “additional explained variance” refers to adj. R^2^ from which the base model adj. R^2^ has been subtracted.

Further linear mixed effect modelling details can be found in Supplementary Information.

#### PermANOVA

To model the whole metabolite matrix on a set of determinants for comparison purposes to a previous publication, we used the adonis2 function from the vegan R package with 1000 permutations and the “euclidean” method. Since only baseline metabolomics data was assessed, we included samples without follow-up measurement, increasing sample size to n = 2803 study participants of whom the respective data was collected.

(For other methods used for supplementary figures see Supplementary Information).

### Role of funders

The funders had no role in study design, data collection, data analyses, interpretation, or writing of this report.

## Results

### Metabolome principal components differ in their biochemical composition

**Fig. 1:**
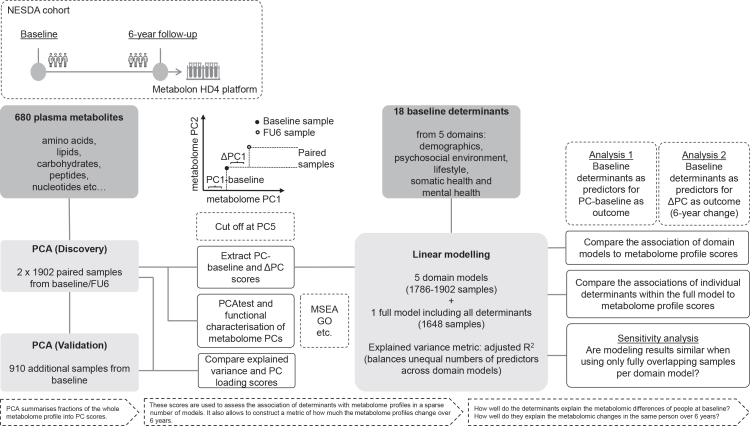
Study design and workflow (n = 1902 for main analysis). PCA–Principal Component Analysis, FU6–six-year follow-up, MSEA–Metabolite Set Enrichment Analysis, GO–Gene ontology analysis.

**Fig. 2:**
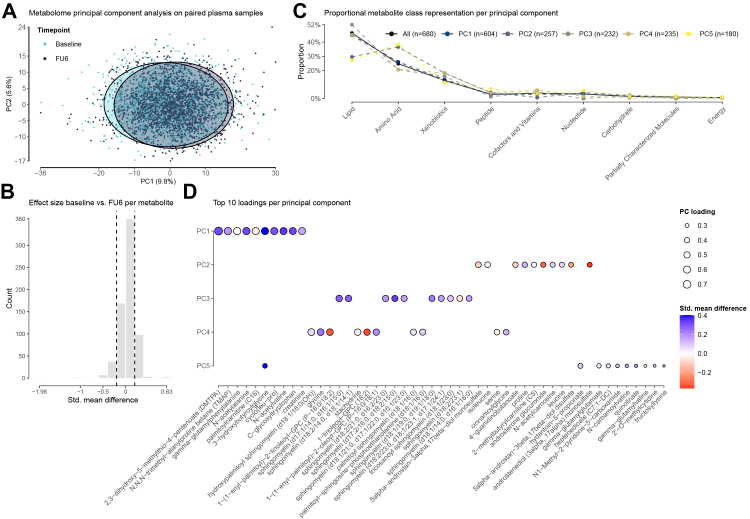
Description of the first five metabolome PCs. **A** In a PCA plot using PC1 and PC2, the samples from baseline and six-year follow-up (FU6) display a shifted metabolomic state (the two density circles do not fully overlap). **B** All 680 metabolites’ standardised mean difference (SMD) for baseline versus FU6 shown as a histogram. Dashed lines mark SMD = ±0.2. Count refers to number of metabolites. **C** After a PCA test, the significant loadings (metabolites) per PC are categorised by metabolite class. A bootstrapping approach was used to determine significance. While PC1 metabolites do not show different metabolite class proportions compared to all measured metabolites, PC2-5 display divergent proportions for several metabolite classes, especially lipids and amino acids. **D** Metabolites with highest loading scores per principal component. A higher loading score indicates more importance for the respective PC score.

To characterise the metabolome principal components of our study participants, we first performed a principal component analysis (PCA) on 3804 paired samples from 1902 subjects and 680 metabolite levels (Fig. 1, Supplementary Table S2). We observed a shift of the FU6 samples compared to the baseline samples in the dimensionality-reduced metabolome space (Fig. 2A). To enhance the relevance and interpretability of our findings, we retained PC1 to PC5 because these together captured a high proportion (27%) of total variance between samples (Supplementary Fig. S2A). Inspection of the scree plot demonstrated an elbow at PC5, after which additional PCs only contributed minimally (<3% per PC) to the total explained variance. Beyond PC5, incremental increases were negligible and did not meaningfully improve representation of the underlying data. To validate the underlying PC structure, we conducted a separate PCA on 910 held-out baseline samples for which no follow-up measurement was available (Supplementary Table S3). We correlated the 680 metabolite loading scores from the first PCA (discovery) with those from the second PCA (validation) and determined moderate to high Pearson’s correlations for the respective loading scores (PC1: *r* = −0.94, PC2: *r* = 0.96, PC3: *r* = −0.94, PC4: *r* = −0.82, PC5: r = 0.58), confirming that the analysed dimensionality-reduced data captures meaningful and consistent information (Supplementary Fig. S2B). Likewise, the explained variance per PC from the discovery PCA could be confirmed using the validation samples (Supplementary Fig. S2A). When considering change over six years, PC1 and PC3 showed the strongest change, although of small size, between the two timepoints with standardised mean differences (SMD) of −0.28 and 0.25 (Supplementary Fig. S2C). After conducting a PCA test, 604 out of 680 metabolites (88%) significantly contributed to PC1 scores, whereas 257 (37%), 232 (34%), 235 (34%) and 180 (26%) metabolites significantly contributed to PC2,3,4 and 5, respectively (Supplementary Table S4). Unique or overlapping metabolites from all five PCs are shown in Supplementary Fig. S2D. In addition to the broad six year changes observable in PCA, we assessed the stability of metabolite levels between the two timepoints individually: Population-level changes higher than absolute SMD > 0.2 were not common, shown by only 22% of metabolites (Fig. 2B), with moderate within-subject correlation (ICC_median_ = 0.482). Interestingly, in individual paired t-tests we observed a strong reduction in per- and polyfluoroalkyl substances (PFAS) over six years and a striking increase in argininate, dibutyl sulfosuccinate and sarcosine. Dehydroepiandrosterone sulphate and pregnenolone sulphate (neurosteroids known to decrease with age) were also among the metabolites with the highest reduction over six years (Supplementary Table S5 and and Supplementary Fig. S3). These longitudinal metabolite-specific results withstood adjustment for age, sex and shipment in a linear mixed model using the full sample (Supplementary Table S6).

We next aimed to describe the significant metabolites per PC in terms of class and importance: While PC1 metabolites were a general reflection of the overall composition of measured metabolites, we further observed distinct metabolite proportions across the other components: PC2 was characterised by decreased lipids and cofactors/vitamins alongside increased amino acids and xenobiotics. PC3 showed an enrichment in lipids and xenobiotics but depletion in amino acids, peptides, and nucleotides. PC4 exhibited reduced amino acids yet elevated xenobiotics and cofactors/vitamins. Finally, PC5 displayed lower lipid content while showing higher levels of amino acids, peptides, and nucleotides (Fig. 2C). The top ten metabolites with the highest loading scores also varied per PC and are shown in Fig. 2D. The highest loading metabolites were 2,3-dihydroxy-5-methylthio-4-pentenoate (DMTPA) for PC1, 5α-androstan-3α,17β-diol monosulfate for PC2, sphingomyelin (d17:1/14:0, d16:1/15:0) for PC3, hydroxy palmitoyl sphingomyelin (d18:1/16:0(OH)) for PC4, and 3-hydroxybutyroylglycine for PC5.

In summary, we reduced the dimensionality of each participant’s metabolome to five principal component scores, which collectively explain 27% of the total population variance and represent specific biochemical profiles.

### Metabolome PCs show distinct functional enrichments

**Fig. 3:**
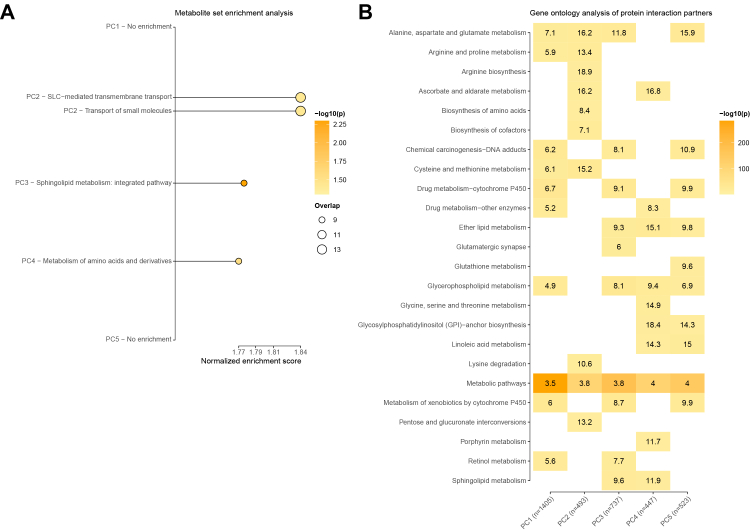
Functional characterisation of the first five metabolome PCs. **A** Ranking all metabolites per PC by their loading score resulted in five lists used as input for metabolite set enrichment analysis (MSEA) in WebgestaltR. Here, all significant RampDB terms are shown. Statistical significance is then estimated via a permutation-based null distribution, and results are corrected using a false discovery rate. PC1 and 5 did not show any significant enrichment of metabolite pathways at the top of the ranked input list. The size of the circles refers to the number of overlapping metabolites from the input list and the respective RampDB set. **B** Results from GO analysis of all available HMDB-derived protein interaction partners of significant PC metabolites using ShinyGO. Shown are only the top 10 results per PC. ShinyGO calculates *P*-values using the hypergeometric test, and false discovery rates are computed via the Benjamini-Hochberg method to correct for multiple testing. Numbers in the grid signify fold enrichment scores (higher fold enrichment ∼ higher coverage of KEGG set by protein input list).

Next, we investigated whether the described metabolome PCs represent concrete biological functions. Metabolite set enrichment analysis (MSEA) on the five sorted lists of metabolome PC loadings revealed that PC2 metabolites with high loadings were significantly enriched for “Transport of small molecules“ and “SLC-mediated transmembrane transport” (protein-mediated transfer of molecules between the extra- and intracellular space) while PC3 and PC4 metabolites with high loadings respectively belonged to “Sphingolipid metabolism: integrated pathway” (involved in cell membrane formation, apoptosis and T-cell function) and “Metabolism of amino acids and derivatives”. MSEA revealed no significant results for PC1 or PC5 (Fig. 3A, Supplementary Fig. S4A). Since MSEA required use of PubChem IDs which were missing in 19–29% of cases per PC, we also used the sub-pathways assigned to each metabolite by Metabolon Inc. to retain all metabolites. We found high proportions (>6%) of metabolites belonging to “Leucine, Isoleucine and Valine metabolism”, “Food component/plant”, and “Androgenic steroids” for PC2, “Sphingomyelins” and “Phosphatidylcholines” for PC3, and “Sphingomyelins”, “Food component/Plant” and “Benzoate metabolism” for PC4. Again, neither PC1 nor PC5 showed strong enrichments in any sub-pathway (Supplementary Fig. S4B). Further, we performed gene ontology analysis (GO) on all Human Metabolome Database (HMDB) documented protein interaction partners of the metabolites per PC. Of note, 13–25% of metabolites could not be used for protein interaction partner retrieval due to missing HMDB IDs (Supplementary Table S7). Most PCs showed unique pathways not observed among the top 10 hits for the others (Fig. 3B, Supplementary Table S8): PC2 metabolite protein interaction partners showed unique enrichments in “Arginine biosynthesis”, “Biosynthesis of amino acids”, “Biosynthesis of cofactors”, “Lysine degradation” and “Pentose and glucuronate interconversions”, while PC3, 4 and 5 showed unique enrichments for “Glutamatergic synapse”, “Glycine, serine and threonine metabolism”/“Porphyrin metabolism” and “Glutathione metabolism”, respectively. Since pathway enrichment results often depend on the choice of the analytical tool, we finally repeated our analysis using weighted gene co-network analysis (WGCNA) and over-representation analysis (ORA) (see Supplementary Information), and saw that our results on “Transport of small molecules”, “Alanine, aspartate and glutamate metabolism”, “Ascorbate and aldarate metabolism”, “Biosynthesis of cofactors”, “Cysteine and methionine metabolism”, “Metabolic pathways” and “Sphingolipid metabolism” replicated (Supplementary Fig. S4C).

Overall, this functional characterisation showed that the plasma metabolome can be divided into principal components that capture metabolite sets representative of distinct biochemical processes. These components can therefore be used as informative outcomes for further linear modelling to assess determinants of the plasma metabolome.

### Demographics and somatic health have the strongest associations with metabolome PCs, cross-sectionally and longitudinally

Having established the biological significance of metabolome PCs, we next aimed to identify baseline determinants of the baseline metabolome and of metabolomic changes over six years (Table 1). We extracted five metabolome baseline scores for PC1-5 and calculated five metabolome change scores termed ΔPC1-5, which were used as model outcomes.

**Table 1:** Dutch cohort characteristics and determinant metrics.

Domain	Determinants	Level	Value	Missing
Demographic	Sex	Male	658 (34.6%)	0 (0%)
		Female	1244 (65.4%)	
	Age (years)		44 [18, 65]	0 (0%)
	Education (years)		12 [5, 18]	0 (0%)
	Degree of urbanisation	Not urbanised	104 (5.5%)	0 (0%)
		Hardly urbanised	143 (7.5%)	
		Moderately urbanised	287 (15.1%)	
		Strongly urbanised	245 (12.9%)	
		Extremely urbanised	1123 (59%)	
Psychosocial	Partner	No	570 (30%)	0 (0%)
		Yes	1332 (70%)	
	Work related stress	No work	430 (22.6%)	144 (7.6%)
		No high strain work	1121 (58.9%)	
		High strain work	207 (10.9%)	
	Leisure activity score		14 [5, 26]	24 (1.3%)
	Daily hassles score		30 [20, 64]	19 (1%)
	Childhood trauma score		0 [0, 8]	4 (0.2%)
	N recent negative life events		0 [0, 7]	0 (0%)
Lifestyle	Alcohol intake (drinks per week)		3.8 [0, 66]	17 (0.9%)
	Smoking status	Never smoker	561 (29.5%)	0 (0%)
		Former smoker	680 (35.8%)	
		Current smoker	661 (34.8%)	
	Physical activity (MET-minutes per week)		2856 [0, 19,278]	111 (5.8%)
Somatic health	N somatic diseases		1 [0, 7]	0 (0%)
	N frequent medications		1 [0, 10]	0 (0%)
	BMI		24.5 [14.7, 55.8]	1 (0.1%)
Mental health	Depression	No diagnosis	1247 (65.6%)	0 (0%)
		Depression diagnosis	655 (34.4%)	
	Anxiety	No diagnosis	1166 (61.3%)	0 (0%)
		Anxiety diagnosis	736 (38.7%)	

Note: For categorical variables, values are N (%); for continuous variables, values are Median [Min, Max].

**Fig. 4:**
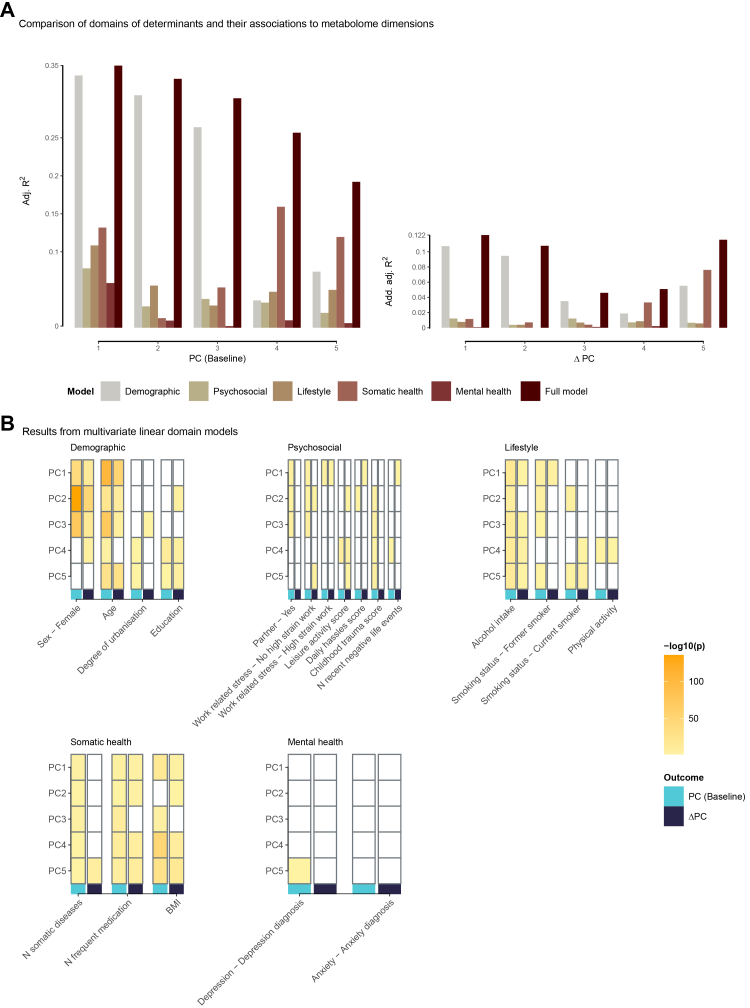
Linear modelling results from the domain models. **A** Comparison of adjusted R^2^ values from linear regression using the domain specific and full models with baseline determinants, with PC-baseline or ΔPC score as outcomes. **B** Visual overview of significant baseline determinants per domain model. For detailed t-statistics see Supplementary Information.

First, we assessed whether any domain of determinants (demographics, psychosocial environment, lifestyle, somatic health, and mental health) explained metabolome PCs or longitudinal changes better than the others. The demographic and somatic health domains explained most variance in PC1 and PC4, respectively (R^2^ = 0.33 and 0.16, Fig. 4A). The lifestyle domain explained most variance for PC1 (R^2^ = 0.11). For the psychosocial and mental health domain, we observed the highest R^2^ of 0.08 and 0.06 also in PC1. The longitudinal analyses revealed generally weaker associations: We saw that the demographic domain also explained most additional variance for ΔPC1 scores (adj. R^2^ = 0.11 in addition to the base model including only PC-baseline and shipment batch), while the somatic health domain explained most additional variance in ΔPC5 scores (additional adj. R^2^ = 0.08). The lifestyle domain explained most additional variance for ΔPC4 scores (additional adj. R^2^ = 0.01, respectively). For the psychosocial and the mental health domain, the highest additional R^2^ of 0.012 and 0.002 were seen for ΔPC1+3 and ΔPC4, respectively. Overall, the highest explained variance was observed using the full model for PC1 (adj. R^2^ = 0.35) and ΔPC1 (additional adj. R^2^ = 0.12). An overview of all significant determinants per domain model and PC is shown in Fig. 4B. In brief, all the 18 chosen determinants showed significant associations across (Δ)PC1-5, with *anxiety diagnosis* being the only exception. An *anxiety diagnosis* showed nominally significant associations in the adjusted mental health models, but it did not withstand Benjamini-Hochberg (BH) correction. As an alternative to the BH correction, we also conducted a stepwise Benjamini-Bogomolov (BB) correction,^50^ which fits the chosen domain-wise testing approach. In the first step, the mental health domain was excluded due to lacking domain-level statistical significance, thereby losing the significant association between depression and PC5 from the BH set of significant findings from the domain models. In the second BB adjustment step, results for the remaining domains were largely similar to those obtained with BH: seven associations reached significance under BB that did not under BH (four in the lifestyle domain and three in the psychosocial domain), while no BH-significant associations were lost in these domains (Supplementary Table S9).

**Fig. 5:**
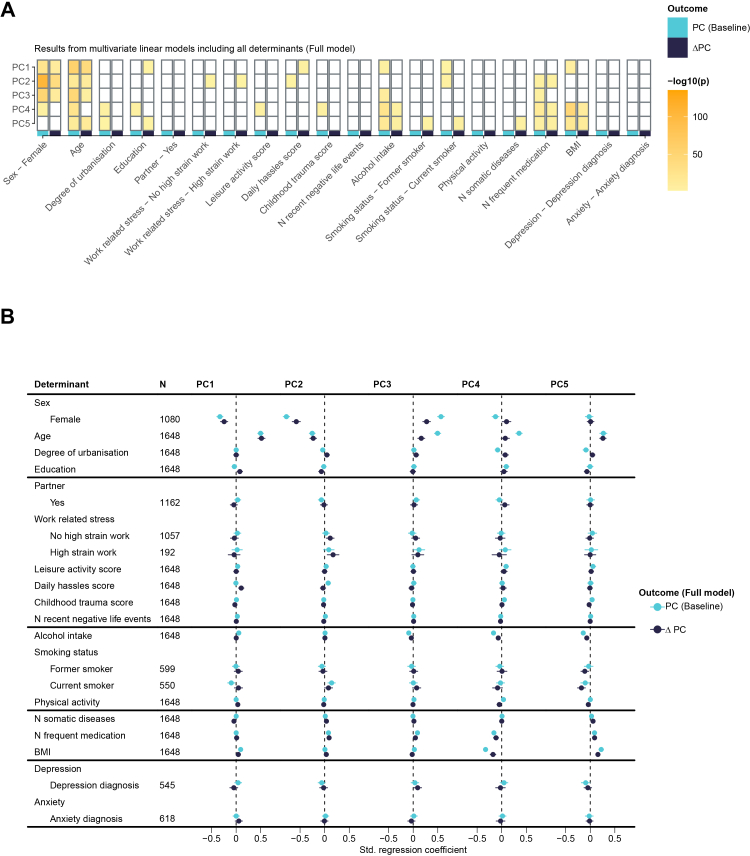
Linear modelling results from the full models. **A** Visual overview of significant baseline determinants. **B** Forest plot of standardised regression coefficients and 95% confidence intervals for all determinants in the full model. For detailed t-statistics see Supplementary Information and Table S10.

As opposed to the separate domain models, the full models provide an overview of the independent associations of all 18 baseline determinants on PC-baseline or ΔPC scores (Fig. 5A and B, Supplementary Table S10). In the following, the individual determinants’ associations with metabolome PCs are listed in order of strong to weak effect sizes. Expectedly, *sex* emerged as one of the most predictive determinants of metabolome PCs at baseline and for six year change, showing associations for PC1-4 and ΔPC1-3. Similarly, *age* was associated with PC1-5 and ΔPC1,2,3,5. The *number of medications* influenced PC2-5 scores and ΔPC2,4,5. *Alcohol intake* was reflected in PC1,3,4,5 and ΔPC4,5. *BMI* showed associations with PC1,4,5 and ΔPC4,5. *Current smoking* was associated with metabolites across PC1-2 and ΔPC5, while *former smoking* showed lasting influences on ΔPC5. *Years of education* were associated with PC4 and ΔPC1,5. *Daily hassles* were associated with PC2 and ΔPC1. *Degree of urbanisation* was linked to PC4,5. *No high strain work* (versus *No work*) and *high strain work* showed associations with ΔPC2. *Leisure activity* and *reported childhood trauma* showed associations with PC4. *Number of somatic diseases* showed an association with ΔPC5. *Partner status*, *physical activity* and a *depression diagnosis* had only nominally significant associations with PC2, ΔPC4,5 and PC5/ΔPC3, respectively, which did not withstand BH correction. An *anxiety diagnosis* and *number of recent negative life events* had no nominally significant associations. All associations between the metabolome PCs, their functional enrichments and significant determinants from the cross-sectional and longitudinal modelling are listed as a summary in Table 2. Moreover, a PermANOVA of *age*, *sex*, *smoking status,* and *BMI* on n = 2803 baseline metabolome measurements revealed that those determinants alone explained 8.9% of variance in our sample (*P* < 0.001).

**Table 2:** Results summary.

Metabolome PC	Metabolite class representation	MSEA results	GO results on protein interaction partners (Top 3, ranked by fold enrichment)	Best domain model for PC-baseline and ΔPC	Significant determinants (Full model for PC-baseline score)	Significant determinants (Full model for ΔPC change score)
PC1 (604 metabolites)	General (similar to overall dataset composition)	n.s.	Alanine aspartate and glutamate metabolism Drug metabolism-cytochrome P450 Chemical carcinogenesis-DNA adducts	Demographic	Sex, Age Alcohol intake, Current smoking BMI	Sex, Age, Education Daily hassles
PC2 (257 metabolites)	Less lipids, more amino acids, more xenobiotics, less cofactors and vitamins than PC1	Transport of small molecules SLC-mediated transmembrane transport	Arginine biosynthesis Alanine aspartate and glutamate metabolism Ascorbate and aldarate metabolism	Demographic	Sex, Age Daily hassles Current smoking Medication	Sex, Age No high strain work, High strain work Medication
PC3 (232 metabolites)	More lipids, less amino acids, more xenobiotics, less peptides, less nucleotides than PC1	Sphingolipid metabolism: integrated pathway	Alanine aspartate and glutamate metabolism Sphingolipid metabolism Ether lipid metabolism	Demographic	Sex, Age Alcohol intake Medication	Sex, Age
PC4 (235 metabolites)	Less amino acids, more xenobiotics, more cofactors and vitamins than PC1	Metabolism of amino acids and derivatives	GPI-anchor biosynthesis Ascorbate and aldarate metabolism Ether lipid metabolism	Somatic health	Sex, Age, Urbanisation, Education Leisure activity, Childhood trauma Alcohol intake Medication, BMI	Alcohol intake Medication, BMI
PC5 (180 metabolites)	Less lipids, more amino acids, more peptides, more nucleotides than PC1	n.s.	Alanine aspartate and glutamate metabolism Linoleic acid metabolism GPI-anchor biosynthesis	Somatic health	Age, Urbanisation Alcohol intake Medication, BMI	Age, Education Alcohol intake, Former smoking, Current smoking Somatic diseases, Medication, BMI

When we expanded our analysis from domain-specific to full models, we observed several changes. Most notably, for PC2, *daily hassles* remained significantly associated while other psychosocial factors became weaker associated or lost statistical significance. A similar attenuation was noted for *physical activity* and *number of recent negative life events* and all PC-baseline associations for *number of somatic diseases*.

Given the strong influence of *age*, *sex* and *BMI* on metabolite levels as seen in the analysis above, additional domain model results adjusted individually for *age*, *sex* and *BMI* can be found in Supplementary Table S11. In general, subsequent results resembled those of the full models, indicating that the domain-model associations between certain determinants and metabolome PCs were attenuated by these important determinants.

Taken together, these results showed that baseline determinants explained a larger proportion of variance in PC-baseline scores as compared to ΔPC scores and that metabolome PC1-5 are differentially associated with the five domains. Across most metabolome PCs, the R^2^ increased by adding information on somatic health and lifestyle beyond demographic characteristics. We further observed that, while the main influences on the metabolome in our study pertained to *sex*, *age*, *alcohol intake*, *BMI* and *medication*, a wide variety of other factors contributed additional but subtler influences. These subtle influences were more abundant in domain models when fewer mediators and confounders were present. Lastly, we observed that determinants linked to the baseline metabolome PCs did not necessarily have significant associations with changes over six years and vice versa.

## Discussion

In this analysis of the plasma metabolome, we examined five metabolome PCs derived from principal component analysis and their associations with participant characteristics across demographic, psychosocial, lifestyle, somatic and mental health domains. Of primary interest, we identified that the main cross-sectional and longitudinal influences on the metabolome pertained to demographic, somatic health, and lifestyle factors, while psychosocial and mental health exposures added only subtle gains for explained variance in metabolome PCs in comparison to the other domains.

Furthermore, principal component analysis, metabolite set enrichment analysis, Metabolon Inc. sub-pathway analysis and WGCNA revealed that metabolome PCs captured distinct biological processes, many of which were conserved across computational approaches. Most importantly, gene ontology analysis on metabolites’ protein-interaction partners reflected potential influences of specific exposures: PC2, primarily influenced by demographic factors (especially sex), showed enrichment in pentose and glucuronate interconversions linked to Uridine 5’-diphospho-glucuronosyltransferase (UGT) enzymes which mediate the inactivation of e.g., oestrogens via glucuronidation. In the domain model analysis, it was PC2 which had the most significant associations with psychosocial factors out of all five PCs, next to PC1. PC4, predominantly shaped by somatic health factors including medication use and especially BMI, was enriched for drug metabolism and porphyrin metabolism, an essential pathway for haemoglobin synthesis, and Glycine, serine and threonine metabolism, a pathway which has been observed for maternal obesity in cord blood and general adiposity.^51^^,^^52^ PC5 showed consistent associations with lifestyle factors and medication and was uniquely enriched for glutathione metabolism, likely reflecting the antioxidant and detoxification demands associated with alcohol consumption, smoking and medication use.^53^

We observed that our chosen determinants explained a limited amount of additional variance in metabolite change over time beyond what is captured by baseline metabolite levels. This is likely due to the restricted range of variability in six year metabolite change in our longitudinal analysis (ICC_median_ = 0.482), which is consistent with previous studies, showing metabolomic stability over several years of follow-up.^4^^,^^19^^,^^54^ Additionally, we might also miss explained variance by modelling only initial baseline exposures rather than accumulated exposures over six years (where applicable). Strikingly, age influenced all metabolome change scores except for ΔPC4. Other studies also found that age had a strong influence on longitudinal metabolomic trajectories.^55^^,^^56^ In fact, the robust association between age and the metabolome might explain why number of somatic diseases and number of recent negative life events–both correlated with age though with different directionality–showed fewer significant independent associations in the full model than in the domain models. Notably, many other determinants in our study showed statistically significant associations with longitudinal metabolomic change in our analysis, but these did not seem to follow a systematic pattern. Education is worth highlighting in this context, as it showed significant longitudinal associations with ΔPC5 in both the domain and full model. Due to its close link to socioeconomic status, this underlines the long-term potential of socioeconomic inequality to associate with metabotype instability in addition to previously observed cross-sectional observations.^57^^,^^58^

When focusing on individual metabolites, we observed strong reductions in PFAS molecules over six years. Moreover, the neurosteroids dehydroepiandrosterone sulphate (DHEA-S) and the closely related pregnenolone sulphate (PS) were also among the most decreasing metabolites over six years. DHEA-S and PS have been investigated for their memory-enhancing and antidepressant effects and were suggested to attenuate memory deficits in Alzheimer’s disease induced by beta-amyloid β25-35.^59^^,^^60^ They have also been discussed as potential therapeutics for several psychiatric conditions.^61^ Therefore, the observed reduction in DHEA-S and PS over six years in our cohort could be linked to ageing phenotypes, although a prominent study has revealed no beneficial effects of DHEA supplementation.^62^ Among the highest increasing compounds, we found argininate, dibutyl sulfosuccinate and sarcosine. The arginine metabolism was shown to be involved in cancer malignancy.^63^ Dibutyl (sodium) sulfosuccinate is a synthetic surfactant used in pharmaceuticals, detergents as well as personal care products.^64^ Contrastingly, sarcosine has previously been found to decrease with age in mice.^65^ Importantly, these results stem from a paired, but unadjusted t-test comparison of baseline and FU6 data including all 1902 participants. However findings for e.g., PFAS and neurosteroids did not change with regards to statistical significance when using the full sample in a linear mixed effects model, including age, sex, and shipment batch as covariates (see Supplementary Information and Table S6). Of note, the timepoint estimates and −log10(*P*) for DHEA-S and pregnenolone sulphate shrank after this adjustment.

Our findings further indicate that metabolome PCs are differentially shaped by the assessed domains, reflecting the notion that exposure domains differentially affect biochemical processes. The most pronounced effects were observable for sex, age (demographics), medication, BMI (somatic health), and lastly alcohol consumption and smoking (lifestyle). These associations align with previous studies and likely occur via interaction with established biological mechanisms,^3^, ^4^, ^5^ including the influence of sex hormones,^66^ ageing and mortality-related metabolomic processes,^56^^,^^67^^,^^68^ microbial metabolite production^69^, ^70^, ^71^ and xenobiotics exposure^72^ which collectively shape the level of circulating metabolites. Our cross-sectional PermANOVA R^2^ estimate of 8.9% for the combined effects of age, sex, BMI, and smoking on the metabolome exceeds previous reports by almost double, although this may partly be explained by differences in metabolite coverage and technical factors.^4^ Interestingly, former smoking demonstrated weaker associations with metabolome PCs than current smoking, suggesting the possibility of metabolomic recovery following smoking cessation. Furthermore, leisure activity had one significant association with PC4 even after adjusting for alcohol intake, smoking, physical activity, and partner status. This suggests an influence of spending free time outdoors, a finding that warrants structured follow-up analyses. While leisure activity and physical activity share some overlapping elements, leisure activity primarily reflects social activity, whereas physical activity captures sports, exercise, and movement. The consistent association of degree of urbanisation with PC4,5 in the demographic domain model and in the full model suggests that exposure to city environments might lead to a metabolomic signature shaped by airborne xenobiotics and toxins, but also lifestyle factors which differ between the rural and the urban population. Similar observations in plasma have been made by a recent Chinese study.^29^

Psychological stress emerged as another contributor to metabolome PCs, with correlations observed across multiple stressors including daily hassles, occupational stress, and childhood trauma. These stress-related traces, while more subtle than those from other domains, may occur through multiple pathways including epigenetic modifications,^73^, ^74^, ^75^, ^76^ altered microbial metabolite production,^77^ or behavioural adaptations affecting body composition and sleep patterns.^78^^,^^79^ Influences of stress on the metabolome have been studied previously but none explicitly considered subclinical occupational or daily stress in a large cohort.^13^ Our findings on childhood trauma and its metabolomic signature recapitulate previous work from NESDA, which showed a dose–response relationship between childhood trauma and specific plasma metabolites.^18^ A depressive disorder diagnosis (MDD and/or dysthymia) was associated in the mental health domain model with metabolites captured in our study by PC5, in line with previous studies which identified correlations between metabolite levels and a depression diagnosis.^9^^,^^80^^,^^81^ This association was reduced and no longer statistically significant in the full model including all determinants, which may suggest that the association between depression and metabolite levels could be explained by relevant factors included in the full model. Nevertheless, it is important to remark that estimates from the full model should be interpreted with caution due to potential overadjustment, as for each determinant all other factors may represent not only confounders to be taken into account but also mediators (e.g., unhealthy lifestyle as a consequence of depression) or colliders (e.g., chronic diseases determined by both depression and metabolomic alterations) that should not be included in the model for proper interpretation.

This study represents a large-scale investigation that simultaneously examines demographic, psychosocial, lifestyle, somatic and mental health determinants of the metabolome using both cross-sectional and longitudinal data. The comprehensive nature of the chosen determinants enables direct comparison of explained variance across domains and provides a basis for more targeted mechanistic investigations. Hence, the novelty of our study lies in its multiplicity, combining high sample size with high metabolite coverage and detailed exposures from two timepoints. However, several limitations warrant consideration. First, due to limited availability of certain determinants at FU6 and limited longitudinal variability of participant characteristics, we decided to omit analyses on how changes in determinants during follow-up associate to metabolomic change over time and thus focused on initial (baseline) exposures. We also acknowledge that our study uses metabolome PCs and exposure domains rather than individual metabolites and exposures. This approach was deliberately chosen to identify broad, hierarchical associations between the exposure domains and the metabolome, offering a comprehensive first exploration that can inform and prioritise follow-up studies targeting specific metabolites and exposures. In line with this we also suggest including more objective exposures in future research, which rely less on self-report.

While we capture a large amount of information on interindividual metabolomic variation at two timepoints using the top five metabolome PCs (27%), we strictly cannot claim full metabolome-wide coverage. However, due to high multiple-testing burden and biological redundancy amongst intercorrelated metabolites, we deemed this approach a more efficient analysis than modelling single-metabolite levels. Furthermore, the reliance on PCA, while facilitating dimensional interpretation, necessarily involves loss of information compared to analyses on a metabolite-level or more than the top five PCs. While we further justify the exclusion of unknown metabolites from our analysis based on their limited potential for replicability in other studies due to their high measurement uncertainty, we acknowledge the fact that they might have increased the explained variance in our analysis. Additionally, HMDB protein interaction partners were used for gene ontology analysis, which also includes connections between proteins and metabolites that have not been validated. Computational enrichment tools such as MSEA or GO can deliver false positives and although we validated biological process terms across several methods, we cannot claim full certainty on whether these would replicate in an independent study. Furthermore, the ethnic, genetic, and geographic homogeneity of our Dutch cohort may limit generalisability to other populations. Finally, untargeted metabolomic analyses are highly sensitive to laboratory protocols, which can introduce noise through anti-clotting agents, storage time, shipment, and handling procedures.^82^^,^^83^ Lastly, missing samples in the full model were not fully at random, which may have potentially affected results compared to domain models. Sensitivity analysis on domain models with overlapping samples led to robust results.

Future research should focus on disentangling the overlapping influences of the here presented determinants on the metabolome, which will help to better understand the mechanisms that can either harm or protect the metabolism. Moreover, follow-up investigations should inspect mediating factors and individual metabolites to identify actionable and specific biomarkers underlying the associations presented here, especially regarding exposure to mental illness and medication/antidepressants, which usually have more specific/targeted effects that are hard to capture with principal components. Previous studies aiming to link metabolomics with depressive and/or anxiety disorders have shown mixed results, indicating that individual metabolites associate with major depressive disorder, but not with anxiety disorders,^8^ or with anxious/distress symptoms within major depressive disorder,^84^ but also with pure anxiety disorders.^85^ It is likely that the chosen analysis approach for this study cannot fully resolve or disentangle these associations in the first five PCs. However, the metabolome PCs proved useful for capturing other factors: PC2 metabolites, for instance, emerged as particularly interesting due to their characteristic pathway enrichment, strong associations with sex and age and associations with psychosocial factors (including psychological stress) in the domain and full models. Comparative analysis of PC2 metabolites between both sexes could enhance our understanding of the molecular mechanisms underlying psychological stress exposures and may inform hypotheses for future prevention research with potential relevance for females, who are at higher risk of developing stress-related diseases such as depression.^86^^,^^87^

In conclusion, this study represents a large-scale epidemiological investigation examining a wide variety of determinants that influence metabolite levels in plasma cross-sectionally and longitudinally. Our approach extends previous research by investigating metabolome PCs rather than individual metabolites, and by examining not only standard factors but also associations with psychosocial and lifestyle factors. Our findings suggest that future longitudinal metabolomic studies may benefit from extending covariate adjustment beyond the conventional set of age, sex, BMI, medication, and smoking. By incorporating additional exposures such as psychosocial factors, model accuracy and interpretability may improve. Through a comprehensive analysis across five conceptual domains, we observed that several underexplored influences (urbanisation, work stress, daily hassles, and leisure activity) appear to associate with the plasma metabolome. These findings inspire new questions of how life exposures are embedded into the metabolome and how these could inform therapeutic interventions or prevention options for conditions involving metabolomic dysregulation.

## Contributors

DK: Conceptualisation, Methodology, Formal analysis, Writing–Original Draft, Visualisation, Writing–Review & Editing; YM: Conceptualisation, Methodology, Writing–Review & Editing, Supervision; LKMH: Conceptualisation, Methodology, Writing–Review & Editing, Supervision; NJAvdW: Writing–Review & Editing; MA: Writing–Review & Editing; CRB: Writing–Review & Editing; SM: Writing–Review & Editing; GK: Data generation, Writing–Review & Editing; RKD: Data generation, Writing–Review & Editing; BWJHP: Data collection and generation, Conceptualisation, Writing–Review & Editing, Supervision. DK, YM and GK have accessed and verified the underlying data. All authors read and approved the final version of the manuscript.

## Data sharing statement

Code is available from Zenodo at https://doi.org/10.5281/zenodo.17869122.

The data used to support the findings of this study are available upon reasonable request from NESDA, Amsterdam: nesda@amsterdamumc.nl. Information on how to request the study data, including the data sharing policy, can be found at https://www.nesda.nl/nesda-english/.

## Declaration of interests

Dr. Kaddurah-Daouk is an inventor on a series of patents on use of metabolomics for the diagnosis and treatment of central nervous system diseases and holds equity in Metabolon Inc., Chymia, and Metabosensor. Drs. Arnold and Kastenmüller are inventors of patents on applications of metabolomics in neurodegenerative/neuropsychiatric diseases and hold equity in Chymia LLC, which had no role in this work. The other authors declare no conflicts of interest.
