# Supplementary material for: Determinants of the plasma metabolome: cross-sectional and longitudinal associations over six years in the NESDA cohort
Source: eBioMedicine. 2026 Jul 23;130:106384. doi: 10.1016/j.ebiom.2026.106384 (PMC13427574; doi:10.1016/j.ebiom.2026.106384)
Supplement: Supplementary Figs. S1–S4 [file mmc1.pptx]

## Slide 1
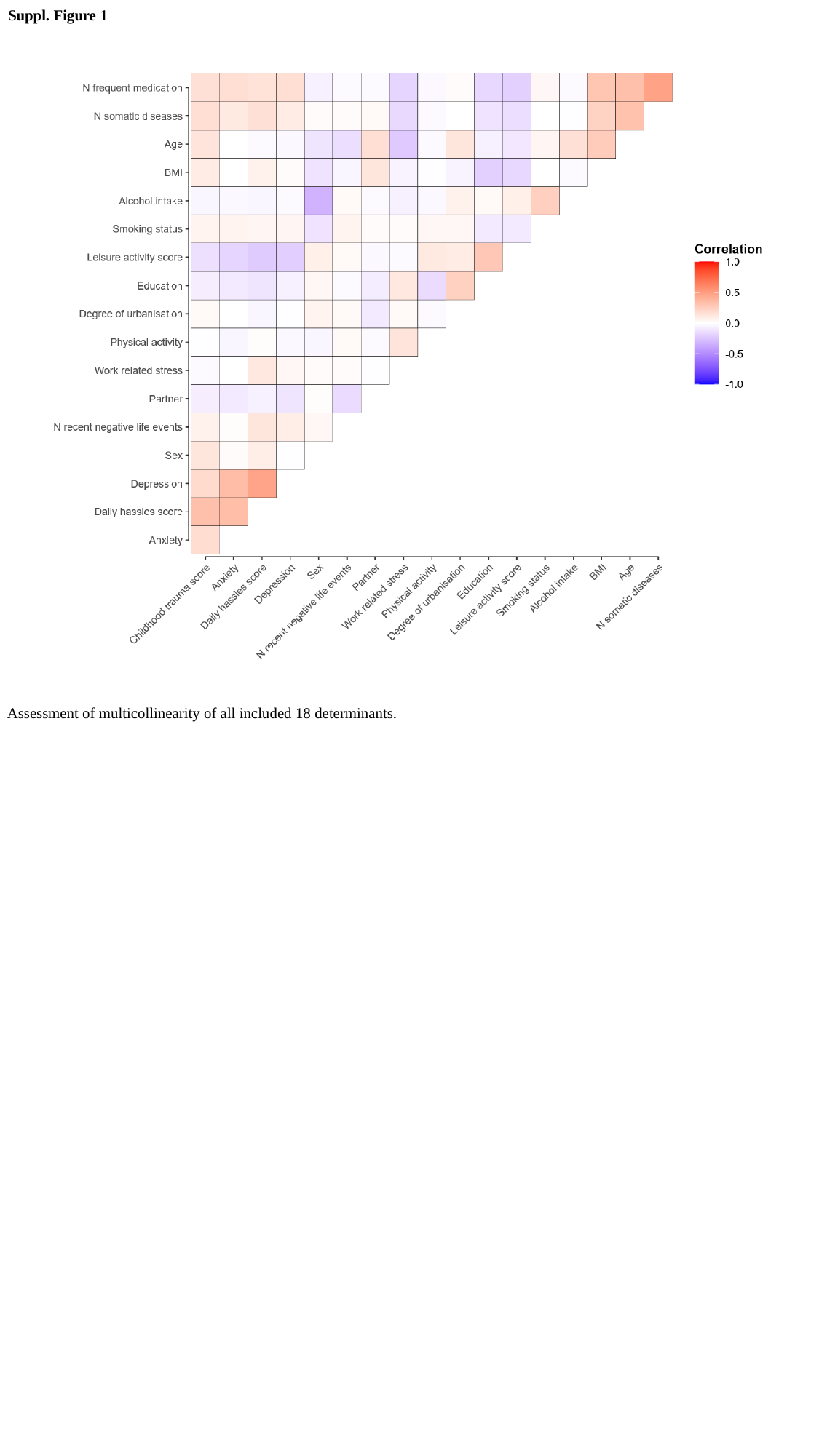

Suppl. Figure 1
Assessment of multicollinearity of all included 18 determinants.

## Slide 2
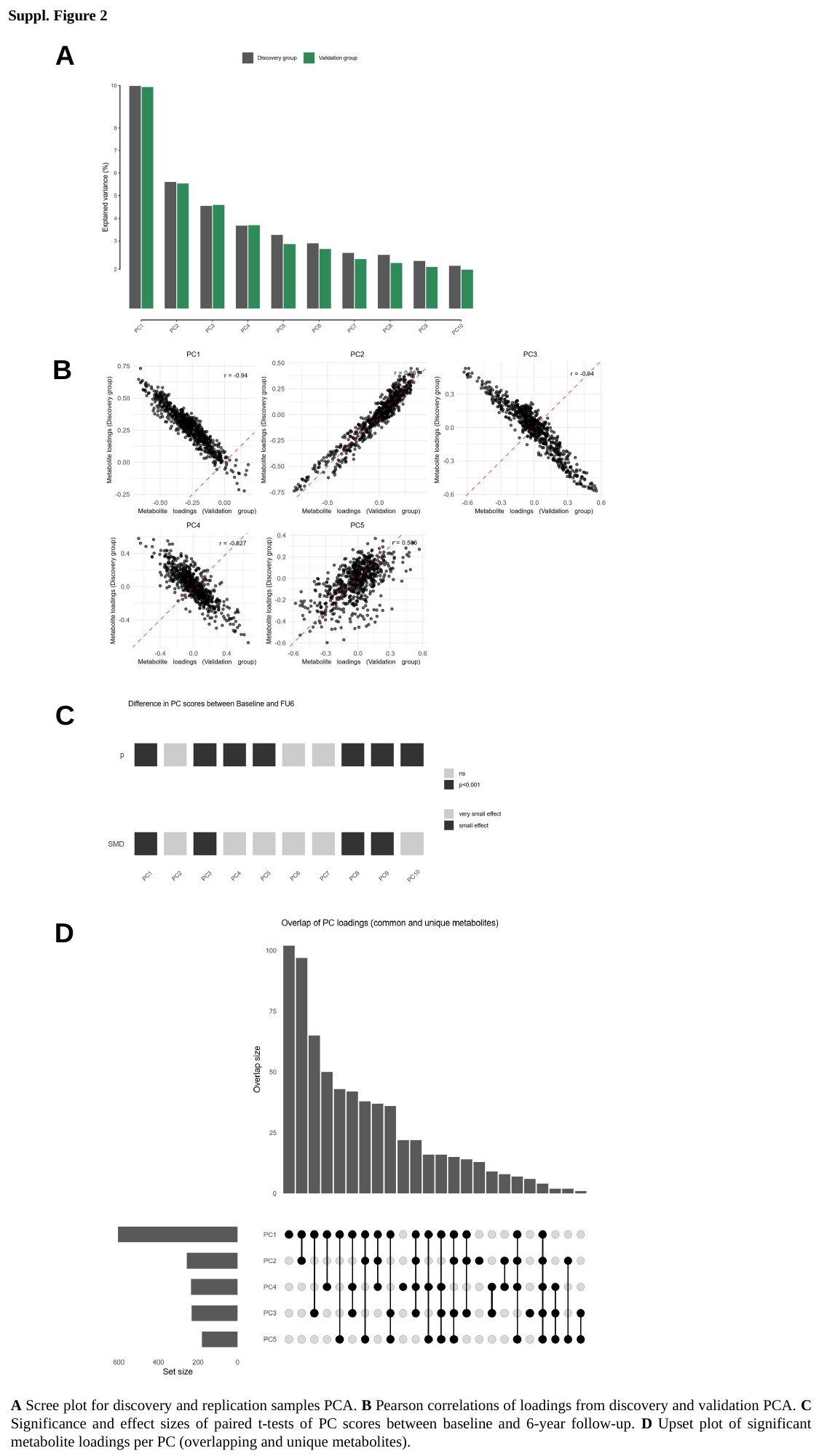

Suppl. Figure 2
A
B
C
D
A Scree plot for discovery and replication samples PCA. B Pearson correlations of loadings from discovery and validation PCA. C Significance and effect sizes of paired t-tests of PC scores between baseline and 6-year follow-up. D Upset plot of significant metabolite loadings per PC (overlapping and unique metabolites).

## Slide 3
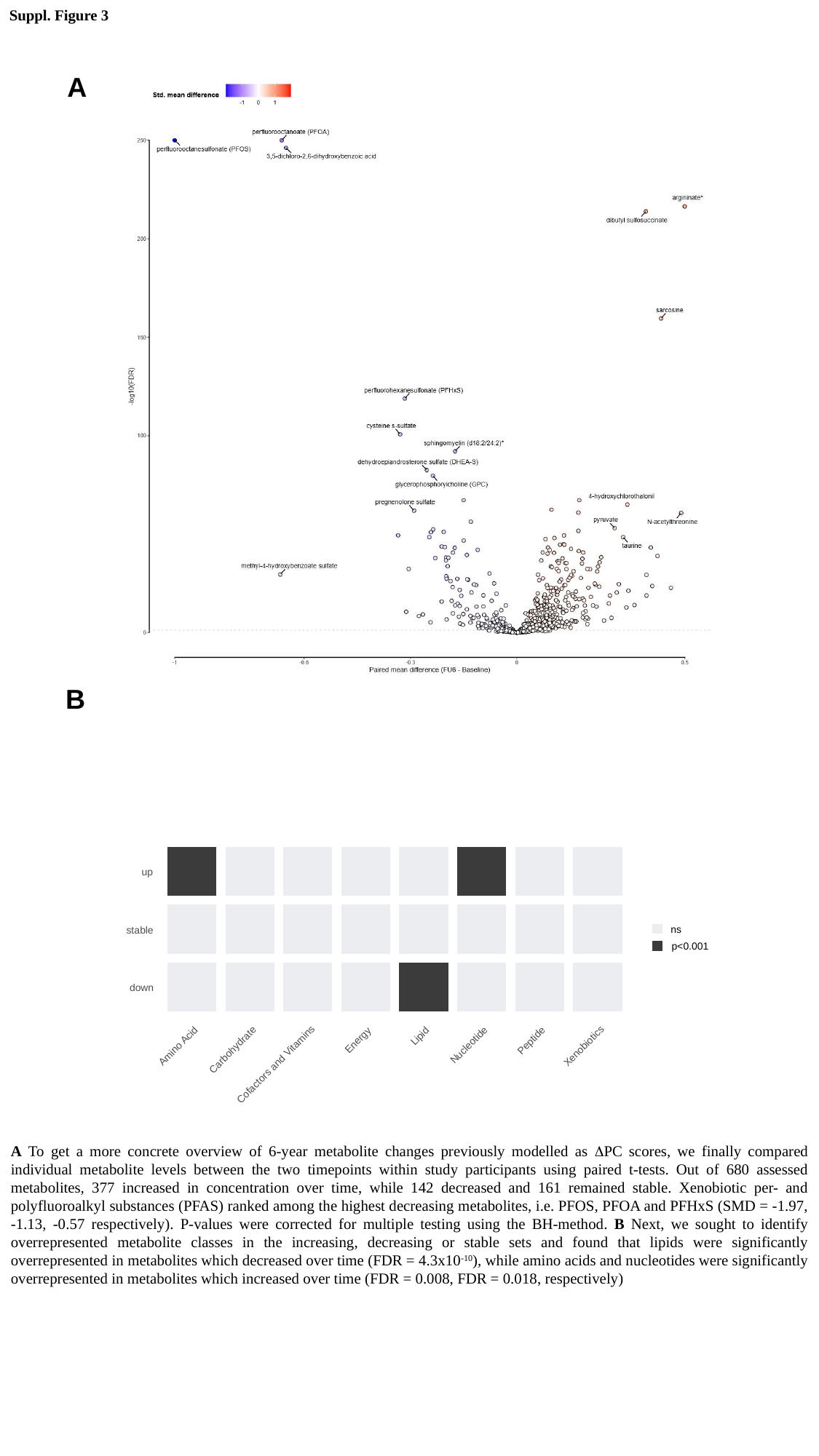

Suppl. Figure 3
A
B
up
ns
stable
p<0.001
down
Lipid
Energy
Peptide
Nucleotide
Amino Acid
Xenobiotics
Carbohydrate
Cofactors and Vitamins
A To get a more concrete overview of 6-year metabolite changes previously modelled as ΔPC scores, we finally compared individual metabolite levels between the two timepoints within study participants using paired t-tests. Out of 680 assessed metabolites, 377 increased in concentration over time, while 142 decreased and 161 remained stable. Xenobiotic per- and polyfluoroalkyl substances (PFAS) ranked among the highest decreasing metabolites, i.e. PFOS, PFOA and PFHxS (SMD = -1.97, -1.13, -0.57 respectively). P-values were corrected for multiple testing using the BH-method. B Next, we sought to identify overrepresented metabolite classes in the increasing, decreasing or stable sets and found that lipids were significantly overrepresented in metabolites which decreased over time (FDR = 4.3x10^-10^), while amino acids and nucleotides were significantly overrepresented in metabolites which increased over time (FDR = 0.008, FDR = 0.018, respectively)

## Slide 4
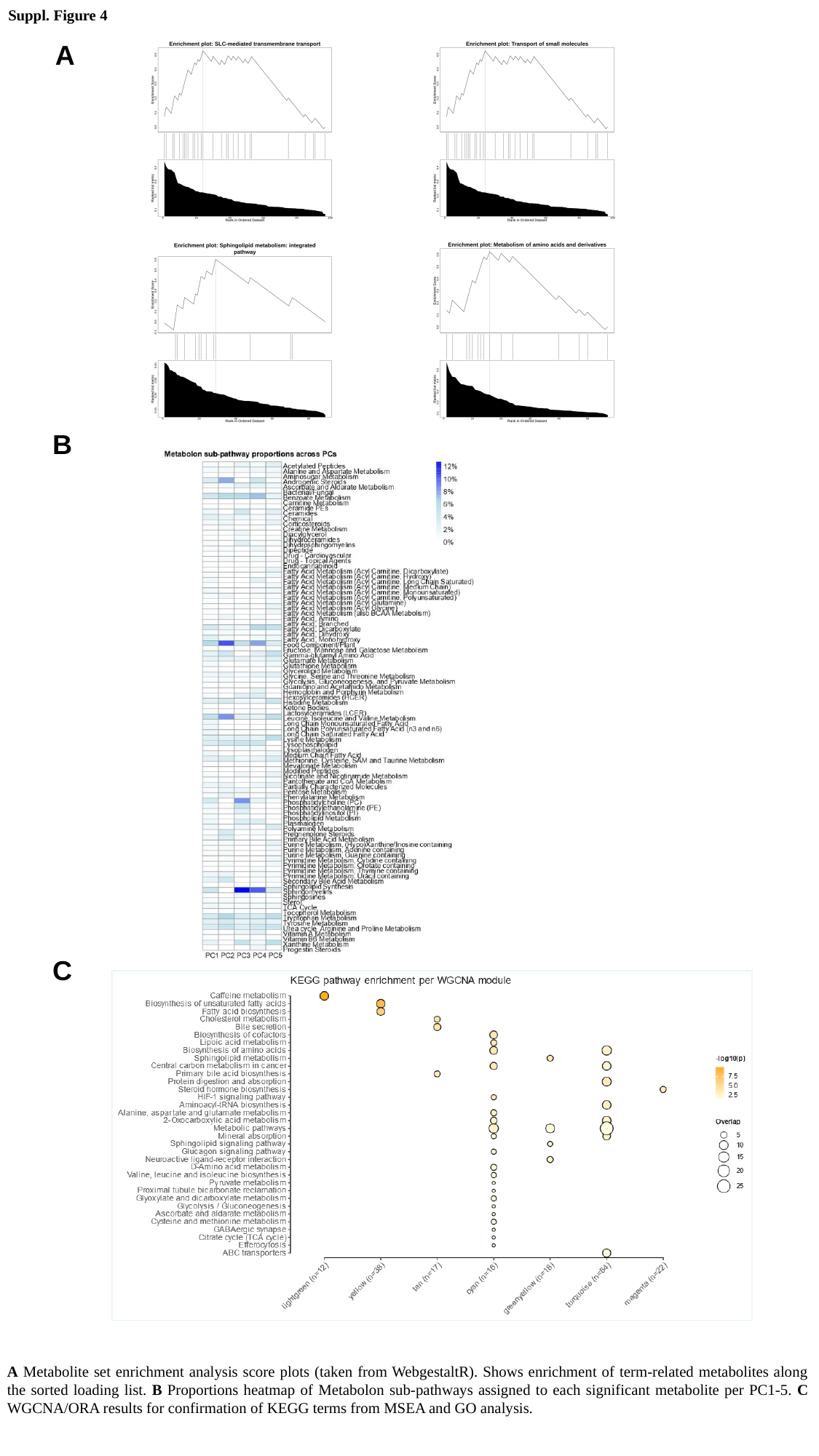

Suppl. Figure 4
A
B
C
A Metabolite set enrichment analysis score plots (taken from WebgestaltR). Shows enrichment of term-related metabolites along the sorted loading list. B Proportions heatmap of Metabolon sub-pathways assigned to each significant metabolite per PC1-5. C WGCNA/ORA results for confirmation of KEGG terms from MSEA and GO analysis.
